# Computational insights for target prioritization and pesticide effects on intrauterine growth restriction (IUGR)

**DOI:** 10.6026/973206300223501

**Published:** 2026-06-30

**Authors:** Adarsh Kumar Shukla, Anuj Kumar Tyagi, Sandeep Kumar

**Affiliations:** 1 Department of Multidisciplinary Research Unit, Dr. Bhimrao Ramji Ambedkar Government Medical College, Kannauj, Uttar Pradesh, India, 209732; 2 Department of Microbiology, Dr. Bhimrao Ramji Ambedkar Government Medical College, Kannauj, Uttar Pradesh, India, 209732; 3 Department of Community Medicine, Autonomous State Medical College, Kaushambi, Uttar Pradesh, India, 212207

**Keywords:** Comparative toxicogenomics, intrauterine growth restriction (IUGR), molecular simulations, placental transcriptomic data, toxicity profiling

## Abstract

Intrauterine growth restriction (IUGR) is a leading cause of maternal and neonatal morbidity and mortality, particularly in low- and middle-income countries. Placental transcriptomics data were analysed to mapped dysregulated pathways via STRING-based PPI networks, identifying hubs and validating pesticide targets by molecular simulations. Rank-based prioritization identified IL7R, LCK and ZAP70 as top hub proteins driving placental dysfunction with cypermethrin exhibiting the lowest binding affinities across them (docking scores: -7.7, -8.7, -9.3 kcal/mol, respectively). Molecular dynamics simulations confirmed the stability of these docked complexes, while toxicity profiling indicated genotoxic potential for permethrin and high aquatic toxicity for deltamethrin. Thus, we show the critical molecular mediators linking pyrethroid exposure to IUGR, suggesting further experimental validation to support maternal health risk assessment.

## Background:

Pregnancy is a complex physiological state that requires careful coordination between the mother and the fetus. The disturbed equilibrium led to pregnancy-related disorders, including preeclampsia, gestational diabetes, anemia, placental abruption, intrauterine growth restriction (IUGR) etc. [1]. Among these disorders, IUGR is a major cause of health problems and death in mothers and newborns worldwide, especially in low- and middle-income countries [2]. Impaired development of the placental blood vessels, which affects the supply of oxygen and nutrients to the fetus, is a key factor that contributes to IUGR [3]. Increasing evidence suggests that problems with gene expression, cell signalling, immune response and metabolic balance contribute too many issues related to pregnancy complications [4]. According to the National Family Health Survey (NFHS-5, 2019-2021), the national rate of preterm births (PTMBs) in India is about 12.4%, it accounts for nearly one-fourth of the global number of PTMBs, with around 3.02 million premature infants born each year [5]. This high rate is linked to several factors, such as maternal malnutrition, anaemia, infections, insufficient antenatal care and socioeconomic differences [6]. In India, exposure to pesticides during pregnancy is becoming more recognized as a significant risk factor for IUGR and other negative outcomes for the fetus, including higher infant mortality [7]. A published study [8] shows that organochlorine pesticides and organophosphates can weaken placental function and fetal growth by causing oxidative stress, cell death and hormonal imbalances in the placenta. A case-control study by Anand and Taneja [9] showed that prenatal exposure to organochlorine pesticides negatively affects fetal growth, resulting in lower birth weight, shorter body length and smaller head circumference. Additionally, exposure to pyrethroids has been linked to reduced fetal growth and an increased risk of small-for-gestational-age births [10]. Di Renzo [11] reported that these pesticides may build up in the placenta, especially during periods when the fetus is rapidly developing and has immature metabolic functions. This accumulation can lead to long-term growth and developmental problems. Recent advancements in RNA sequencing (RNA-Seq) have allowed for detailed profiling of gene expression in various maternal-fetal compartments, such as maternal blood, placental tissue, decidua and fetal membranes [12]. Despite the growing number of available datasets, the molecular mechanisms behind negative pregnancy outcomes are still not fully understood [13]. This lack of understanding highlights the need for comprehensive systems biology approaches that can go beyond studying individual genes. Such approaches can help identify overall regulatory networks and disrupted biological pathways at the maternal-fetal interface [14]. Therefore, it is of interest to identify significantly disrupted biological pathways through published transcriptomic profiling data. It will also create and analyze maternal-fetal interaction networks, including protein-protein interactions and gene co-expression modules [15] and identify key regulatory points that could serve as potential biomarkers or therapeutic targets in maternal-fetal disturbances.

## Material and methods:

## K-means clustering of PPI network:

The IUGR-associated protein targets were used as input for the protein-protein interaction (PPI) network analysis through the STRING database (https://string-db.org/). K-means and hierarchical clustering algorithms (k=3) were used to divide the network into tightly connected groups that represent functional categories. The steps for the K-means clustering algorithm were taken from the studies by Ghazal *et al.* [16] and Ikotun *et al.* [17]. Hierarchical clustering assessed cluster quality using indices like the Jaccard index and modularity. The results were compared across different algorithms to ensure reliability [18]. The K-means clustering algorithm is well-known for its flexibility, efficiency and straightforward application. It is regarded as one of the top 10 clustering methods in data mining [19].

## Gene ontologies of target proteins:

All target genes were analyzed for gene ontology (GO) enrichment across categories like biological process, molecular function and KEGG pathways, using a method similar to that of Shukla and Kukshal [20]. The results were interpreted with precision, gene count and false discovery rate (FDR) as measures of enrichment significance for each GO term.

## Prioritization of hub proteins:

The hub proteins in the PPI network were identified by computing topological properties like node degree and betweenness centrality, by using CytoHubba plugins in Cytoscape platform. Proteins were ranked based on degree and Maximal Clique Centrality (MCC) scoring method. Selected hub proteins as a robust target for subsequent toxicants and docking analysis.

## Pesticides networking of potential exposomes:

The pesticide exposure datasets were obtained from the Comparative Toxicogenomics Database (CTD; https://ctdbase.org), followed the approach of Shukla *et al.* [21]. Using CTD-curated chemical-gene and chemical-disease interactions, association scores were calculated to build integrated exposome-protein networks.

## Molecular docking study:

The three-dimensional (3D) structures of the hub proteins were obtained from the RCSB Protein Data Bank (https://www.rcsb.org/) and AlphaFold (https://alphafold.ebi.ac.uk/), along with their respective PDB IDs. The receptor chains were subsequently prepared following the method described in referenced study [18]. The 3D structures of the lead pesticide molecules were retrieved from the PubChem database (https://pubchem.ncbi.nlm.nih.gov/). Docking protocols for PyRx and AutoDock Vina tool were then established, following Shukla *et al.*[22]. Finally, docking simulations were performed and binding poses were ranked using scoring functions to determine binding affinity and molecular interactions [23].

## Ligand binding site analysis:

The docked complexes with the minimum binding affinity were used as input in BIOVIA Discovery Studio to analysed the bonding patterns between receptor and ligand, following the method described in [24].

## Structural flexibility analysis:

The root mean square fluctuation (RMSF) analysis was performed using the CABS-flex 2.0 web server (http://biocomp.chem.uw.edu.pl/CABSflex2) to assess residue-wise backbone flexibility based on coarse-grained Monte Carlo simulations of protein dynamics. The receptor-ligand docked poses were submitted using default parameters, including auto-generated distance restraints (Cα-Cα/SS2 mode, gap=3 Å, min/max distances 3.8-8.0 Å, rigidity=1), native-state-mimicking temperature and approximately 1000 trajectory models generated from 100,000 total cycles using asymmetric Metropolis sampling for Boltzmann-distributed ensembles.

## Toxicity profiling of lead pesticides:

Toxicity profiling of the lead pesticides was conducted using the pkCSM database (https://biosig.lab.uq.edu.au/pkcsm/).

## Results and discussion:

## Protein-Protein Interaction (PPI) network analysis of IUGR targets with K-means clustering (k=3):

The STRING network analysis of placental transcriptome profiling data in IUGR revealed key protein interactions involved in placental development, highlighting hub proteins that may play central roles in the pathophysiology of IUGR [25]. Furthermore, the constructed PPI network was subjected to K-means clustering with three clusters (K=3), to identify distinct functional modules [18]. The constructed network, depicted in Figure 1 (see PDF), revealed three major clusters with central nodes and associated interacting proteins. Cluster 1, cantered around tumor necrosis factor (TNF), includes key nodes such as IL7, LTB, IL1R, ZAP70, LCK and BCL11B. This cluster highlights a significant involvement of inflammatory and immune signalling pathways, with TNF serving as a central hub connecting multiple immune-related proteins, consistence with previous findings [26]. Cluster 2, anchored by PSME2 (Proteasome Activator Subunit-2), encompasses nodes like C1QTNF1, ARPC2, EVI5 and WARS1. This group of proteins suggests a role in proteasomal regulation and related cellular processes, with PSME2 acting as a pivotal node linking these functions [27]. Cluster 3, cantered on GRN (Granulin Precursor), includes nodes such as SIN3B, PIEZO1, PSG6, PODXL, SCEL, EIF2AK2, ARM2 and CD99L2. This cluster suggests involvement in cell adhesion, signalling and structural organization, with GRN as a central coordinator [28]. The present k-means cluster analysis revealed the heterogeneity of IUGR-related protein interactions, with distinct modules reflecting immune regulation, proteasomal activity and cellular adhesion/signalling, respectively.

## Gene ontology of constructed PPI:

The biological process enrichment analysis of IUGR targets, derived from PPI networks, revealed several key processes with significant involvement. The analysis, summarized in (Figure 2A - see PDF), highlights the principal processes ranked by gene count and false discovery rate (FDR). The positive regulation of biological process was the most enriched category, with a gene count of 25 and an FDR of 3.2e-05, indicating a broad regulatory role in IUGR-related mechanisms. This FDR score of 3.2e-05 was used to identify genes and biomarkers indicating a broad regulatory impact in IUGR mechanisms. The immune system process followed, with a gene count of 15 and an FDR of 8.1e-05, suggesting a substantial contribution of immune-related pathways. Regulation of immune system process and positive regulation of immune system process were also prominent, with gene counts of 10 and FDR values of 2.0e-04 and 5.2e-04, respectively, underscoring the regulatory dynamics of the immune systems in IUGR [25]. Regulation of cell adhesion, regulation of cell-cell adhesion and positive regulation of cell adhesion emerged as significant, with gene counts of 10, 15 and 25, respectively and FDR values ranging from 1.3e-03 to 3.3e-03, highlighting the importance of cellular adhesion in IUGR pathology [25]. Immune response and leukocyte activation were identified with gene counts of 10 and an FDR of 3.3e-03, indicating roles in immune activation. de Boer *et al.* [29] reported that the positive regulation of gene expression with a gene count of 10 and an FDR of 3.3e-03, suggesting transcriptional regulation as a contributing factor. The molecular function enrichment analysis of IUGR targets, derived from protein-protein interaction (PPI) networks, identified key functions with significant involvement. The analysis, summarized in (Figure 2B - see PDF), highlights the principal function ranked by gene count and similarity within groups. Non-membrane spanning protein tyrosine kinase activity was identified as a significant molecular function, with a group similarity score of 0.8, indicating a notable role in enzymatic activity related to IUGR [30]. The KEGG pathway enrichment analysis of IUGR targets, derived from PPI networks, identified several key pathways with significant involvement. The T cell receptor signalling pathway was found to be the most enriched category, with a gene count of 8, indicating a prominent role in immune signalling related to IUGR [31]. The NF-kappa B signalling pathway followed, with a gene count of 3, indicating involvement in inflammatory and immune regulatory processes. The present study also identified primary immunodeficiency with a gene count of 4, pointing to potential immune system deficiencies associated with IUGR. The study indicated that IUGR targets are significantly enriched in processes related to immune regulation, cell adhesion and gene expression, with varying degrees of statistical significance as reflected by the FDR values. The gene ontology study of IUGR targets also highlighted tyrosine kinase activities, with potential implications for cellular signalling pathways.

## Network analysis and ranking of hub proteins:

The present rank-prioritisation analysis revealed interleukin-7 (IL7R), lymphocyte-specific protein tyrosine kinase (LCK) and tyrosine- protein kinase ZAP-70 (ZAP70) as the top three hub proteins with the highest MCC scores, namely 103, 98, 96, respectively (Figure 3A - see PDF). These finding indicated their strong centrality and functional importance within the network (Figure 3B - see PDF). Among these 3 hub proteins, other notable proteins included tumour necrosis factor (TNF), interleukin-2-inducible T-cell kinase (ITK) and B-cell lymphoma/ leukaemia-11B (BCL11B), which also demonstrated high connectivity and ranked among the top 10 highly interacting proteins within this cluster. Interestingly, immune-related molecules dominated the ranking, suggesting that dysregulation of immune signalling pathways may contribute significantly to the pathophysiology of IUGR [32]. In the present PPI network analysis of IUGR, the proteins such as LTB, PSME2 and GRN were also identified among the prioritized targets, although they had comparatively lower MCC scores of 8, 3 and 2 respectively. The prioritization of hub proteins underscores the central involvement of immune and inflammatory pathways in IUGR. The identified hub proteins, particularly IL7R, LCK and ZAP70, may serve as potential biomarkers or therapeutic targets for future studies aimed at understanding and mitigating IUGR-associated complications.

## Study of regional distribution of pesticide in human biological samples across Indian states:

The present study reviewed various classes of pesticides and their distribution in human biological samples and evaluated them across different regions of India. Organophosphate pesticides, including chlorpyrifos, malathion, ethion, profenophos, parathion and monocrotophos, were detected in blood and urine samples collected from populations in Punjab, Telangana and Karnataka [33]. These findings align with previous reports documenting exposure of individuals in these regions to organophosphates, highlight significant pesticide absorption through environmental or occupational routes [34]. The presence of these pesticides in biological fluids underscores the ongoing risk of exposure in agricultural and rural communities within these states. This regional pesticide exposure profile complements data for other pesticide classes such as organochlorines, including dichlorodiphenyltrichloroethane (DDT), dichlorodiphenyl dichloroethylene (DDE), dichlorodiphenyl dichloroethane (DDD), hexachlorocyclohexane (HCH), endosulfan and methoxychlor, which have been identified in blood, urine and milk samples from Punjab, Haryana, Kashmir, Uttar Pradesh, Tamil Nadu, Kerala, Rajasthan, Assam, Madhya Pradesh [35, 36, 37]. Human exposure to pesticides was found to differ between urban Kolkata and semi-urban Nadia regions in West Bengal, India. Pyrethroids were detected in human milk samples in India for the first time. This finding suggests a shift in pesticide usage patterns in India from organochlorines to pyrethroids [7]. The results revealed the important evidence of the systemic toxicity of multiple pesticide classes in human populations across India and underline the need for future research to mitigate maternal health risks associated with pesticide exposure.

## Comparative toxicogenomic study:

The regional reported toxicants and their associated disease mapping analysis was done with the utilization of the CTD. Several pesticides were identified as high-priority toxicants potentially associated with IUGR and adverse developmental outcomes. Chlorpyrifos ranked as the top toxicant, with a substantial number of reported studies (n=82) linking its exposure to craniofacial abnormalities, followed by associations with delayed prenatal exposure effects (n=18), prenatal injuries (n=28) and spontaneous abortion (n=7). Endosulfan emerged as another major hazardous pesticide, with 62 studies implicating it in craniofacial abnormalities and 11 studies reporting delayed effects from prenatal exposure. Methoxychlor and cypermethrin were also retrieved as significant maternal health-related toxicants, recorded in 50 and 21 studies, respectively, for their association with craniofacial abnormalities. The mapping of these toxicants and corresponding study distributions is presented in Figure 4 (see PDF).

## Molecular docking study:

The present molecular docking study evaluated the binding affinities of selected vulnerable pesticides against three prioritized hub target proteins related to IUGR such as IL7R, LCK and ZAP-70. Cypermethrin exhibited the strongest binding affinity across all three receptors, with docking scores of -7.7, -8.7 and -9.3 kcal/mol, respectively. The obtained docking scores suggests that cypermethrin has highly stable interaction with these hub targets [15]. Similarly, permethrin was found to be second most strongly interacting pesticide with the docking scores of -7.3, -7.9 and -9.1 kcal/mol against IL7R, LCK and ZAP-70, respectively. Binding affinities of -7.5, -8.4 and -8.8 kcal/mol were observed for cyhalothrin, which also showed favourable interactions against the hub teins IL7R, LCK and ZAP-70, respectively [38]. DDT and DDE also showed considerable binding affinities of -7.1, -7.9 and -7.2 kcal/mol and -6.6, -7.6 and -6.3 kcal/mol, respectively, values close to those of cypermethrin, reinforcing their potential biological relevance as modulators of these proteins. Another pesticide, Carbendazim, showed good interactions, with binding scores, ranging from -5.5 to -7.4 kcal/mol, indicating moderate but still favourable interactions [23]. The compound, chlorpyrifos, had the weakest docking scores, particularly against IL7R (-4.7 kcal/mol), suggesting relatively low binding stability with the receptor proteins tested. The docking scores (Figure 5 - see PDF) reflect the predicted free energy changes upon ligand binding, with more negative values indicating stronger and more stable interactions [22]. These comparative results provide crucial insights for prioritizing compounds for experimental validation in toxicological or mechanistic studies, guiding further structural optimization to enhance binding efficacy and specificity. Thus, based on the present docking analysis, cypermethrin, permethrin and cyhalothrin were identified as the lead compounds for further binding pattern analysis, followed by DDT and DDE, whereas chlorpyrifos appears less promising in the context of these targets.

## Binding residues pattern analysis:

The docking score of cypermethrin demonstrated a robust binding affinity toward the ZAP-70 hub protein through a diverse array of non- covalent interactions, as illustrated in Figure 6 (see PDF). This binding pattern revealed that cypermethrin is stabilized in the binding pocket of ZAP-70 by multiple van der Waals interactions involving residues such as ASN348, ARG465, PHE349, LEU482, ASP461 and PHE516. These van der Waals interactions contribute distance-dependent attractive forces between ligand and receptor atoms, optimizing binding pose and affinity score [39]. Additionally, significant alkyl and Π (pi)-alkyl interactions with PRO502, LEU486 and TYR506 contribute to enhanced hydrophobic and steric compatibility [18]. The ligand also engages in specific Π-anion and Π-cation interactions with ASP461 and ARG460, respectively, highlighting the role of electrostatic forces in enhancing binding specificity [38]. The observed bond distances and interaction patterns confirm strong and multifaceted ligand-protein recognition, suggesting that cypermethrin can modulate ZAP-70 conformation and potentially interfere with its normal signalling cascades [40]. Residues such as ARG119, PRO2, LEU43, TYR69, LEU118, GLY44, ARG63, SER602, LYS151, LYS603, GLN234 and ALA154 of the ZAP-70 hub protein, highlighted the importance of hydrophobic stabilization in the binding of cyhalothrin, as depicted in Figure 7 (see PDF). In addition, notable alkyl and Π-alkyl interactions are established with residues such as PRO4, VAL604, ARG119, ILE153, MET122 and LEU43, demonstrating hydrophobic compatibility and steric fit within the active site [41]. The interaction map also revealed one Π-sulfur interaction with residue MET122 (yellow in colour) which indicated specificity and strength in this docked association [42]. The aromatic moiety of cyhalothrin was involved a carbon-hydrogen bond and Π-alkyl contacts, supporting multi-point attachment and stable complex formation. These findings indicated that cyhalothrin achieves robust and multifaceted binding with ZAP-70, integrating van der Waals, hydrophobic, Π-alkyl and Π-sulfur interactions to promote a strong and specific cyhalothrin ZAP-70 protein interaction. The present study also revealed that permethrin interacts strongly with the ZAP-70 hub protein, as depicted in Figure 8 (see PDF). The binding pocket analysis demonstrated extensive van der Waals contacts with residues such as ARG465, ASN466, ASN348, LEU482, LEU486, SER478, ASP479, MET414, LEU468, GLY418, GLY420, PRO421, LYS369 and ALA367, providing hydrophobic stabilization for permethrin within the ZAP-70 protein. Additionally, multiple Π-alkyl interactions occur with VAL352, GLY347, PHE349, LEU344 and CYS346, enhancing steric compatibility and ligand anchoring through aromatic and alkyl side-chain engagement [43]. Permethrin also showed strong Π-anion interactions with ASP461 and ASP479, indicating electrostatic complementarity and binding specificity of the ligand [39]. Notably, one halogen (fluorine) bond with ALA417 and carbon-hydrogen bonds further enrich the binding landscape [44]. In this docked pose, the aromatic rings of permethrin engaged in Π-Π T-shaped and amide-Π stacked interactions, reinforcing ligand stability and specificity within the protein interface [38]. This composite interaction pattern highlights a robust and specific ligand-protein association, suggesting that Permethrin may substantially influence ZAP-70 activity through direct interaction with multiple key residues in the hub domain.

## RMSF analysis:

The RMSF analysis was conducted using the CABS-flex 2.0 server to characterize the residue-wise flexibility of the ZAP-70 protein in complexes with cypermethrin, cyhalothrin and permethrin. As shown in Figure 9 (see PDF), permethrin induced the highest degree of fluctuation in several regions, with prominent peaks above 4.5 Å observed around residues 110 and 280, indicating significant local mobility [45]. Cyhalothrin resulted in intermediate flexibility, especially within residues 250-320 and 500-560, with RMSF values generally ranging between 3.5 and 4.5 Å. In the case of cypermethrin, the lowest overall fluctuations were observed, predominantly below 3 Å, indicating enhanced protein stability [46]. The residue interval between 200 and 320 remained highly flexible in all complexes, while several terminal and loop regions, such as residues 1-20 and 50-599, also displayed heightened RMSF values. These findings suggest that stable interactions were maintained in the docked regions. The present RMSF study revealed that each pyrethroid ligand distinctly modulates the protein's dynamic landscape, with permethrin imparting greater segmental mobility and cypermethrin promoting structural rigidity [20].

## Toxicity profiling:

A comparative study of the toxicity profiles of the predicted inhibitors, cypermethrin, permethrin, cyhalothrin and deltamethrin, revealed both commonalities and notable differences in their risk patterns for humans and ecological systems. Data from in silico toxicological predictions showed that all four pesticides generally lack hepatotoxic and skin-sensitizing effects and were not predicted to block cardiac hERG channels, reducing concerns about these specific human health endpoints. This finding aligns with much of the published literature [47, 48], which indicated that relatively favourable acute toxicity profile in mammals for pyrethroids as a class. According to the Holynska-Iwan & Szewczyk-Golec [49], permethrin was also found to adversely affect fertility, immune function, cardiovascular and hepatic metabolism and enzymatic activity. Deltamethrin triggers inflammation, nephrotoxicity and hepatotoxicity while altering antioxidant enzyme activity in tissues. Alpha- cypermethrin impairs immunity and elevates blood glucose and lipid levels. Permethrin stands out as the only compound to test positive in the AMES mutagenicity assay, suggesting a potential for genotoxic effects [50]. This concern echoes emerging epidemiological data linking pyrethroid exposure to possible long-term health risks, although the evidence remains limited and largely associative. In terms of maximum tolerated human doses, permethrin also exhibits the highest margin, whereas deltamethrin has the lowest, reflecting relative variations in potency and possibly differential metabolic clearance reported for these compounds in both humans and animals. In the case of acute oral toxicity (LD50 in rats), cyhalothrin demonstrated the highest LD50 value, 3.386 mol/kg, indicating the lowest acute toxicity, whereas deltamethrin (LD50: 2.967 mol/kg) was the most potent. This trend consistent with earlier comparative toxicological studies [19]. Chronic oral toxicity estimates (LOAEL values) may reflect similar trends, with permethrin (1.507 log mg/kg_bw/day) and cypermethrin (1.501 log mg/kg_bw/day) requiring higher cumulative dosages to elicit chronic adverse effects compared cyhalothrin and deltamethrin. Ecotoxicological endpoints highlighted a major environmental concern: Deltamethrin showed the greatest toxicity to aquatic species, particularly fish (minnow toxicity: -3.022 log mM), substantiating well-established findings that pyrethroids, although generally less hazardous to mammals, pose significant threats to non-target aquatic organisms, often via runoff and leaching into water bodies [51]. This pattern of selective toxicity supports regulatory caution regarding pyrethroid application near surface waters. Overall, the toxicity profiles of these pyrethroids, as presented in Table 1 reflect their distinct risk-benefit balances: low to moderate acute mammalian toxicity and minimal sensitization or hepatotoxicity, but a pronounced genotoxic risk for permethrin and significant aquatic toxicity, especially for deltamethrin.

## Conclusion:

Cypermethrin exhibited the strongest binding affinity to IL7R, LCK and ZAP70 hub proteins in IUGR, followed by permethrin and cyhalothrin, indicating stable ligand-protein interactions. Toxicity profiling revealed low-to-moderate acute mammalian toxicity with limited hepatotoxicity or sensitization for these pyrethroids; however, permethrin posed genotoxic risk and deltamethrin showed high aquatic toxicity. These findings highlight pyrethroid adversity in IUGR, underscoring the need for experimental validation and targeted environmental risk mitigation.

## Advancement to knowledge:

The present findings predicted pyrethroids is biomarkers for IUGR with use of computational approaches.

## Statements and declarations:

## Author contribution statement: 

Adarsh Kumar Shukla contributed to the conceptualization, investigation, data curation and original draft preparation of this study. Anuj Kumar Tyagi contributed to the study conceptualization, resources, proofreading, validation and supervision and Sandeep Kumar provided statistical support.

## Ethical approval statement: 

The study was approved by the Institutional Ethics Committee of Dr. Bhimrao Ramji Ambedkar Government Medical College, Kannauj, on November 24, 2025.

## Patient consent statement: 

This computational study did not involve human samples; therefore, patient consent was not applicable.

## Data availability: 

The data that support the findings of this study are available from the corresponding author upon reasonable request.

## Funding source: 

This work was funded by the Multidisciplinary Research Unit, Dr. Bhimrao Ramji Ambedkar Government Medical College Project (No. MRU/010).

## Figures and Tables

**Table 1:** *In silico* ADMET and toxicity profiles by utilizing the SMILES strings of predicted lead pyrethroids (cypermethrin, permethrin, cyhalothrin and deltamethrin) through pkCSM online tool. Data include AMES mutagenicity; maximum tolerated human dose, hERG I/II inhibition, oral rat acute toxicity, chronic toxicity, hepatotoxicity and skin sensitization, *Tetrahymena pyriformis* toxicity and fathead minnow LC50.

	**AMES toxicity**	**Max. tolerated dose (human) in (log mg/kg/day)**	**hERG I inhibitor**	**hERG II inhibitor**	**Oral Rat Acute Toxicity (LD50) in (mol/kg)**	**Oral Rat Chronic Toxicity (LOAEL) in (log mg/kg_bw/day)**	**Hepatotoxicity**	**Skin Sensitisation**	***T. Pyriformis* toxicity in (log ug/L)**	**Minnow toxicity in (log mM)**
Cypermethrin	No	0.678	No	No	3.284	1.501	No	No	0.487	-2.273
Permethrin	Yes	0.84	No	No	3.134	1.507	No	No	0.487	-2.291
Cyhalothrin	No	0.595	No	No	3.386	1.2	No	No	0.439	-2.126
Deltamethrin	No	0.543	No	No	2.967	1.237	No	No	0.383	-3.022
